# Taxonomy is the cornerstone of biodiversity conservation-SEABRI reports on biological surveys in Southeast Asia

**DOI:** 10.24272/j.issn.2095-8137.2017.061

**Published:** 2017-09-18

**Authors:** Shu-Qiang Li, Rui-Chang Quan

**Affiliations:** ^1^Southeast Asia Biodiversity Research Institute, Chinese Academy of Sciences, Yezin Nay Pyi Taw 05282, Myanmar; ^2^Institute of Zoology, Chinese Academy of Sciences, Beijing 100101, China; ^3^Center for Integrative Conservation, Xishuangbanna Tropical Botanical Garden, Chinese Academy of Sciences, Mengla Yunnan 666303, China

The Southeast Asia Biodiversity Research Institute (SEABRI) is an international scientific research and education organization affiliated directly to the Chinese Academy of Sciences (CAS) and managed by the Xishuangbanna Tropical Botanical Garden (XTBG). By harnessing its connections with all CAS institutes, local institutes and international agencies and leveraging on their resources, it seeks to make a significant contribution to biodiversity conservation in Southeast Asia. SEABRI intends to project itself as a crucial networking platform for biodiversity researchers from both China and Southeast Asia, an internationally respected research and conservation institution on Southeast Asian biodiversity, and a model on cooperation on research and education between China and Southeast Asian countries.

This special issue of *Zoological Research*, entitled "***SEABRI Reports on Biological Surveys in Southeast Asia***" represents a new effort by SEABRI to promote awareness of the biodiversity and its conservation in the region. Because nature conservation is built upon a foundation of knowledge about life¡¯s diversity itself, we are focusing on taxonomic discoveries in the first instance. Seven articles in this issue involve the description of new species from biological surveys in the region. They include a new rain-pool frog (Anura: Dicroglossidae) from Thailand, a new species of snake (Serpentes: Colubridae) and a sisorid catfish (Siluriformes, Sisoridae) from southern China, a new species of landhopper (Amphipoda, Talitridae) from Myanmar, new species of spiders (Araneae, Scytodidae) from China and Thailand, and the discovery of cyprinid fishes (Teleostei: Cypriniformes) in Myanmar. Results of ornithological explorations in northern Myanmar and the phylogenetics of leaf muntjac are also reported. All studies are financially supported by the CAS (2015CASEABRI005, Y4ZK111B01).

Southeast Asia has arguably the highest degree of endemism and species richness in the world. The region has four overlapping biodiversity hotspots: Indo-Burma, Philippines, Sundaland and Wallacea (Myers et al., 2000; Sodihi et al., 2004). This pattern of species richness has been related to plate tectonic events and climate changes (Renema et al., 2008; Leprieur et al., 2016). During the Tertiary, the collision of India with Eurasia resulted in the uplifting of the Himalayas and the Tibetan Plateau, and the extrusion of Indochina (Royden et al., 2008; Hou et al., 2011; Hou & Li, 2017). Geological events probably also triggered climate change, which, in turn, led to an increase in biodiversity in Southeast Asia (Che et al., 2010; Luo & Li, 2017). In this issue, the distribution pattern of land-hoppers in high mountains, which are closely related to the sand hoppers in the seashore, highlights the significance of geography in shaping evolutionary history. The authors (Zheng & Hou, 2017) hypothesize that the collision between India and Eurasia has driven the marine-originated animals to colonize terrestrial habitats, and move to high elevations during the mountain building.

Taxonomic discoveries go beyond just setting the framework for our understanding of the evolution of life on earth. In fact, taxonomy is the cornerstone of conservation, as we need to inventorize our species diversity before we can devise policies to protect them and manage their habitats and our other natural resources in a sustainable way. It is perhaps time to better integrate the science of taxonomy with conservation practices to meet one of the most serious challenges that threaten our planet today. Many species will become extinct before we know that they even exist in Southeast Asia. We must step up our efforts to discover, describe, and document our natural heritage for our future generations. We must provide science-based data to our conservationists, resource managers and policy makers so that they can all pitch in to save our biota. It is a daunting task, but it is necessary, and absolutely urgent.
